# Early Postoperative Parathormone and Calcium as Prognostic Factors for Postoperative Hypocalcemia

**DOI:** 10.3390/jcm11092389

**Published:** 2022-04-24

**Authors:** Anna Daskalaki, Sofia Xenaki, Konstantinos Lasithiotakis, Alexandros Chrysos, Marilena Kampa, George Notas, Emmanuel Chrysos

**Affiliations:** 1 Department of General Surgery, University Hospital of Heraklion, 715 00 Heraklion, Greece; sofiabecks@yahoo.gr (S.X.); kwstaslasith@gmail.com (K.L.); achrysos@ukaachen.de (A.C.); chrysose@uoc.gr (E.C.); 2 Laboratory of Experimental Endocrinology, Department of Laboratory Medicine, University of Crete School of Medicine, 715 00 Giofirakia, Greece; kampam@uoc.gr; 3 Department of General, Visceral and Transplantation Surgery, University Clinic RWTH Aachen, 52074 Aachen, Germany; 4 Department of Emergency Medicine, University Hospital of Heraklion, 715 00 Heraklion, Greece

**Keywords:** hypocalcemia, thyroidectomy, post-surgery, parathormone, calcium

## Abstract

Background. Postoperative hypocalcemia is one of the most common complications after total thyroidectomy. Parathormone (PTH) and calcium levels, measured several hours after surgery, have been suggested as valuable markers for detecting patients at risk for post-thyroidectomy hypocalcemia. We aimed to determine if early post-surgery PTH and calcium levels can be used for the early identification of patients at risk for symptomatic hypocalcemia. Methods. PTH and calcium were measured before surgery and at 10 min and 4 h post-thyroidectomy, in 77 patients. Performance characteristics of PTH and calcium levels and their post/pre-surgery ratios were calculated. Results. Four-hour calcium was a sensitive (93.75%) but not specific (67.61%) indicator of patients at risk for symptomatic hypocalcemia. The 4-h/pre-surgery PTH ratio was the most accurate (90.81%) and the most specific (94.37%) test to identify patients at risk. Serum calcium at 4-h, 4-h/pre-surgery PTH ratio, and PTH at 10 min post-surgery had the higher diagnostic odds ratios (50.86, 32.85, and 29.04, respectively). The 4-h/pre-surgery PTH ratio also had the highest (0.694) Youden’s J statistic. Conclusions. Low serum calcium levels 4 h after thyroidectomy and the 4-h/pre-surgery PTH ratio could be valuable additions to everyday clinical practice in post-thyroidectomy patients.

## 1. Introduction

The most common postoperative complication of total thyroidectomy is hypocalcemia [1]. Post-thyroidectomy hypocalcemia is attributed to the reduction of the functioning parathyroid parenchyma. Intraoperative manipulations may lead to dysfunction of the parathyroid arteries and ischemia of the parathyroid glands, obstruction of the parathyroid venous outflow, thermal trauma, and failure to recognize parathyroid glands, leading to their accidental excision. In contrast, unintentional removal and parathyroid vascular damage combinations seem to be the most common mechanism [2]. In surgery for Graves’ disease, hypocalcemia may also be secondary to hungry bone syndrome [3], with a mechanism similar to hypocalcemia after parathyroid surgery for primary or tertiary hyperparathyroidism [4].

Hypocalcemia due to low parathyroid hormone (PTH) levels can be classified as transient if it lasts less than six months or permanent if it persists for more than six months [5]. There is also wide variability in the reported postoperative hypocalcemia rate due to the lack of a uniform definition of the condition in published studies. Indeed, most studies use solely biochemical criteria, some use exclusively compatible patient symptoms, and some use both. At the same time, the duration of the hypoparathyroidism that defines permanency (6 months, one year, or two years) is another area for debate. However, most studies use six months as a limit point [2]. According to the fifth National Audit Report of the British Association of Endocrine and Thyroid Surgeons, transient postsurgical hypoparathyroidism may appear in up to 24% of patients after thyroidectomy for multinodular goiter or graves [6]. However, in some studies, transient hypocalcemia has been reported in up to 50% of patients [7]. Unfortunately, hypoparathyroidism may be permanent in about 6–12% of these patients [8]. At the same time, this proportion could be higher in patients operated with a total thyroidectomy for cancer due to the more aggressive surgical approach [9]. Clinical manifestations of hypocalcemia are primarily neurological and cardiological and include perioral and acral paresthesias, muscle cramps, tetany, seizures, a prolonged QT interval, and congestive heart failure. Hypocalcemia symptoms increase patient morbidity, and severe hypocalcemia may be fatal. Even transient hypocalcemia is an important reason for patient and physician concern due to the fear for the development of permanent hypoparathyroidism, which could lead to prolonged hospitalization and a reduced quality of life [2]. Therefore, accurate prediction of the risk for postoperative hypoparathyroidism can improve management strategies [1].

Normal blood calcium levels are a prerequisite for the safe discharge of post-thyroidectomy patients from the controlled hospital environment. Pre-emptive prolongation of patient hospitalization after the operation extends patient discomfort. It is not a cost-effective approach, since most patients do not develop clinical symptoms or laboratory-confirmed hypoparathyroidism [10]. Therefore, strategies that may predict patients at risk for post-thyroidectomy symptomatic hypocalcemia could be useful. A decline in serum calcium from its preoperative values at 24 h has been reported to have a high sensitivity for predicting transient hypocalcemia. However, parathyroid hormone levels allow a more direct and precise determination of the long-term function of the parathyroid glands [5,10].

This study aimed to investigate the role of early postoperative PTH and calcium blood levels and post/pre-surgery PTH and calcium ratios as predictors for the development of symptomatic hypocalcemia within 24 h post thyroidectomy.

## 2. Materials and Methods

### 2.1. Patients

The study protocol was approved by the University Hospital of Heraklion bioethics committee (P.N.17439/2021). Inclusion criteria were patients undergoing thyroidectomy at the Department of General Surgery of the University Hospital of Heraklion between November 2017 and February 2020. Eighty-seven patients were enrolled in the study. Exclusion criteria included renal failure, known parathyroid gland dysfunction, and lack of ability to provide informed consent.

Patient data collected included demographics (age, sex, etc.), comorbidities, preoperative diagnosis, pre-surgery thyroid hormonal status, postoperative complications (including clinical symptoms of hypocalcemia or laboratory findings of hypocalcemia, surgical site infection, vocal cord dysfunction, etc.), and pathology findings including the number of parathyroid glands identified in the surgical specimen.

After providing informed consent, preoperative PTH and calcium were measured once 2–7 days before the operation. All patients underwent a total thyroidectomy with Intra-operative Neuro-Monitoring, energy-based cauterization devices, LigaSure, and sutures. However, only sutures were used for vessel ligation in proximity to the laryngeal nerve and the parathyroid glands, since energy-based cautery devices and LigaSure might affect parathyroid vascularization and function due to the spread of terminal energy for at least 4 mm. Parathyroid glands recognized during the operation were left in situ, while any parathyroid gland accidentally removed or appearing devascularized was auto-transplanted into the neck muscles. New blood samples were collected at 10 min and 4 h after completing the surgical procedure.

Patients were considered to have hypocalcemia symptoms if they presented numbness and positive Chvosteck and/or Trousseau signs within the first 24 h after surgery. Any calcium supplementation was performed after samples for PTH and Calcium were collected.

### 2.2. Measurements

Blood samples were collected with a frozen syringe, transferred to ice-chilled no-anticoagulant-containing vials; transported to the lab on ice racks; and cryo-centrifuged at 4 °C, 150 g for 10 min, to allow serum separation. The Advia 1800 Chemistry System (Siemens Healthineers, Erlangen, Germany) was used for the analysis of calcium and albumin levels in all serum samples, while PTH was measured with an Abbott Architect i2000SR or an Abbott Alinity series analyzer with chemiluminescence (Abbott, Abbott Park, IL, USA).

Local laboratory normal values for calcium were 8.2 to 10.6 mg/dL (2.05 to 2.64 mmol/L) and for PTH 12 to 65 pg/mL (1.27 to 6.89 pmol/L). Calcium values were corrected using the formula: serum Calcium + 0.8 × (4 − serum Albumin).

### 2.3. Statistical Analysis

Data were analyzed with IBM SPSS Statistics for Windows, Version 21.0 (Armonk, NY, USA). Demographics were compared with a chi-square and *t*-test, as indicated with the Bonferroni correction for multiple comparisons. Values before and after surgery were compared with a paired *t*-test. The ratios of calcium at 10 min and 4 h over pre-surgery calcium and PTH at 10 min and 4 h over pre-surgery PTH were also calculated and included in the study. Optimal cutoff values were derived from receiver operating characteristic (ROC) curves using the Yuden Index. Correlations were studied with Pearson’s correlation or Spearman’s Rho, depending on the data characteristics. Sensitivity, Specificity, False Negative Rate (FNR), False Positive Rate (FPR), Positive and Negative Predictive Values (PPV, NPV), Positive and Negative Likelihood Ratios (LR+, LR−), and Diagnostic Odds Ratios (DOR) were calculated. A binary logistic regression analysis with single parameters or their combinations was also performed.

## 3. Results

Eighty-seven patients were included in the study. Patient Characteristics and differences between patients who presented hypocalcemia symptoms and patients who did not are presented in Table 1. A neck dissection was not performed on any of these patients.

Sixteen patients (18.4%) presented hypoparathyroidism, with only one (1.2%) developing permanent hypoparathyroidism. Fifteen transient hypocalcemia patients received oral supplementation for less than six months, while 71 did not receive calcium supplementation at discharge. There was no difference in the incidence or onset of hypocalcemia between patients operated for benign or malignant conditions. Personal physicians or endocrinologists undertook the patient supplementation follow-ups.

Four patients with calcium levels between 7.5 and 8.2 mg/dL (1.87 to 2.05 mmol/L) at 4 h after surgery received oral supplementation of calcium carbonate and alfacalcidol, regardless of if they had symptoms or not. Two of them developed symptoms of hypocalcemia at some point during the first 24 h after surgery and were treated with intravenous Calcium supplementation and oral calcium. Twelve patients with calcium between 7.0 and 7.5 mg/dL (1.75 to 1.87 mmol/L) at 4 h after surgery received oral supplementation plus intravenous calcium. No patient presented with clinical symptoms of hypocalcemia while their calcium levels were within the normal range. The response to treatment was monitored, and patients were discharged when their corrected calcium was within the normal range after discontinuation of intravenous supplementation for 24 h. The median duration of hospitalization was two days for patients that did not present hypocalcemia and five days for those who presented hypocalcemia (median hospitalization duration for the whole cohort was three days).

Both patient groups had significantly lower PTH and calcium values after surgery. However, patients who presented postoperative hypocalcemia had significantly lower PTH and Calcium 10 min and 4 h after surgery. They also had significantly lower PTH at 10 min/PTH preoperatively and PTH at 4 h/PTH preoperatively ratios.

Characteristics of the different indices studied for the prognosis of symptomatic hypocalcemia within the first 24 h are presented in Table 2.

Serum calcium at 4 h was the most sensitive test to identify patients at risk of developing symptomatic hypocalcemia (93.75%) with a cutoff of 8.85 mg/dL (2.21 mmol/L). However, this indicator had a low specificity of 67.61%. The 4-h PTH to pre-surgery PTH ratio was the most accurate (90.81%) and the most specific (94.37%) test to identify patients at risk of developing symptomatic hypocalcemia. Interestingly, PTH levels below 30.5 pg/mL (3.23 pmol/L) at 10 min post-surgery had the highest positive likelihood ratio to identify patients at risk for symptomatic Hypocalcemia (LR+ 9.76), while the ratio of 4-h PTH to pre-surgery PTH displayed the second-best positive likelihood ratio (LR+ 8.70). The three tests with the highest diagnostic odds ratio were calcium at 4 h (DOR 50.86), 4-h PTH to pre-surgery PTH ratio (DOR 32.85), and PTH at 10 min post-surgery (DOR 29.04). The ratio of 4-h PTH to pre-surgery PTH also had the highest (0.694) Youden’s J statistic (sensitivity + specificity-1), which is a valuable indicator for the performance of dichotomous diagnostic tests.

Finally, we ran a binary logistic regression analysis with hypocalcemia as the primary endpoint using the above parameters and the Forward Likelihood Ratio Method. In this model, only preoperative PTH, Calcium at 4 h, and the 4-h to pre-surgery PTH ratio were retained as significant (Table 3). As expected, higher values of these parameters were related to the decreased likelihood for hypocalcemia.

## 4. Discussion

Post-thyroidectomy hypocalcemia is the most common adverse event related to this procedure. The most common risk factors for the development of post-thyroidectomy hypocalcemia are a low number (<100) of thyroidectomies performed per year in a medical center, age above 60 years old, female sex [8], and performance of a lymphadenectomy as part of the procedure [9]. It is a major concern for the surgeon and an important issue for patients, ranging from plain discomfort to a major traumatic experience.

Several strategies have been studied regarding the early identification of patients at risk for postoperative hypocalcemia, including PTH and calcium measurements from 10 min to 24 h. We tried to validate the hypothesis that preoperative and early postoperative calcium and parathyroid hormone levels can be used as markers for the safe discontinuation of inpatient monitoring after a total thyroidectomy. Since early identification of patients at risk for symptomatic hypocalcemia is crucial, in this real-world study, we focused on the performance characteristics of PTH and calcium levels before surgery and at 10 min and 4 h post-surgery, as well as their pre/post-surgery ratios.

Our findings suggest that serum calcium levels above 8.85 mg/dL (2.21 mmol/L) 4 h post-thyroidectomy can identify patients at low risk for symptomatic hypocalcemia. On the other hand, the 4-h PTH to pre-surgery PTH ratio with a cutoff point around 0.385 is an excellent indicator for identifying patients at risk. Therefore, a blood sample drawn 4 h after surgery could be valuable for evaluating patients post-thyroidectomy.

Several studies suggest that a within-the-normal-range PTH preoperatively and a low PTH in the early postoperative period can identify patients at risk for developing hypocalcemia after thyroidectomy [11,12,13,14,15,16,17,18].

Most studies, though, focus on next-day measurements. For example, Inversini et al. [13] reviewed 260 thyroidectomies and found that a postoperative PTH level 24 h after surgery below 10.0 pg/mL (1.06 pmol/L) is probably the most accurate (76.0%) and the most specific (83.0%) test for predicting postoperative hypocalcemia. Similarly, Karatzanis et al. [18] analyzed 100 thyroidectomies and reported that a next-day postoperative decrease in PTH by 56% has a specificity and sensitivity of 80% to identify patients at risk for postoperative hypocalcemia. In a study of 143 patients undergoing total thyroidectomy, Cayo et al. [16] evaluated predictive factors that could identify patients at the highest risk for postoperative hypocalcemia and hypoparathyroidism to appropriately tailor postoperative calcium and vitamin D supplementation. The authors concluded that a PTH level less than 10 pg/mL (1.06 pmol/L) on postoperative day one had a sensitivity of 86% and a negative predictive value of 90% for symptomatic hypocalcemia. Finally, in a very recent study by Paladino et al., the largest so far, including 477 total thyroidectomies, the research team used calcium values above 8.66 and PTH values above 44.3 pg/mL (4.70 pmol/L) to identify patients at a low risk for symptomatic hypocalcemia. They proposed an algorithm that could allow the discharge of up to 70% of patients in their cohort without the risk of hypocalcemia or overtreatment [17].

Other groups, like ours, focused on much earlier identification of patients at risk, since this could lead to pre-emptive therapy and decreased patient discomfort. Similar to our findings, Barczyksni et al. [19] reported that using PTH levels below a certain level (10 pg/mL, 1.06 pmol/L) 4 h after surgery was the optimal strategy for predicting low serum calcium. However, the choice of the 10 pg/mL (1.06 pmol/L) cutoff was arbitrary. The significant difference with our optimal PTH cutoff of about 30 pg/mL (3.18 pmol/L) could also be attributed to the different techniques used for the measurement. In a study from Croatia [11], a serum PTH below 27.3 pg/mL (2.90 pmol/L) one hour after surgery had a sensitivity of 77% and a specificity of 71% to identify patients at risk for developing symptomatic hypocalcemia in days 1–5 postoperatively, and these patients needed higher doses of calcium supplements. Gupta et al. [14] analyzed 90 total thyroidectomies and reported that intraoperative PTH starting from 10 min after removal of the thyroid, with a cut off value of 11.3 pg/mL (1.20 pmol/L), was the most accurate predictor for diagnosing postoperative hypoparathyroidism and had the highest sum of sensitivity (91.7%) and specificity (97%) compared to other parameters, a metric similar to the Youden’s J statistic used in our study. Sands et al., instead of focusing on a PTH value, used the percentage of PTH reduction at 1 h after surgery and reported that a decline of 70% or greater is a reliable predictor of hypocalcemia [20]. Based on our experience and the analysis above, ratios (or percentage change from baseline) are easier to interpret and can overcome the barrier of differences in measurements due to the local use of different assays. It is also reasonable to expect that the distinctive characteristics of each patient that place them at the limits of normal distributions could be misleading, as it happens with several other tests. Changes from their baseline are expected to be more accurate indicators of the dynamic changes in calcium and PTH levels after a thyroidectomy.

By allowing thyroid surgeons to identify these patients in the early postoperative period, calcium supplementation can be initiated sooner. Payne et al. even reported that the use of early (1 and 12 h) postoperative PTH could be used for early initiation of therapy and reduction of patients with symptomatic transient hypocalcemia [10]. In an interesting but older metanalysis [21], it was reported that early post-thyroidectomy PTH levels lack the accuracy to predict hypocalcemia and enable early (within 24 h) discharge. However, any PTH measurement from as early as 10 min post-surgery might be used to implement early treatment which will reduce the incidence and the severity of hypocalcemia. Our results are in full accordance with this metanalysis. Since oral calcium supplements may reduce the incidence and severity of post-thyroidectomy hypocalcemia [21], future prospective studies could utilize indicators like the ones we and others suggest to evaluate the potential clinical utility of pre-emptive therapy for hypocalcemia in high-risk patients. If such a study took place, the 4-h PTH to pre-surgery PTH ratio would be an important candidate.

However, our study has several limitations. It was a single-center experience that should be validated in a more extensive study. Preemptive calcium supplementation in four patients, two of whom developed hypocalcemia, suggests that symptomatic hypocalcemia might have been masked in the other two patients. Our cohort had low vitamin-D levels, which can at least partially explain the relatively high levels of preoperative PTH. Our study did not include children where the risk for postoperative hypoparathyroidism is related to younger age, while permanent hypoparathyroidism is related to an older age [22]. Finally, the selection of 10 min and 4 h after surgery for our post-surgery sampling was based on previous studies. Still, these time points might not be optimal for such an application. A study with more time points could provide further insight on the proper timing of post-surgery sampling.

In conclusion, our findings suggest that low serum calcium levels 4 h after thyroidectomy and the 4-h PTH to pre-surgery PTH ratio could be valuable additions to everyday clinical practice in post-thyroidectomy patients.

## Figures and Tables

**Table 1:** Patient characteristics based on the presentation of hypocalcemia or not.

	All Patients	No Hypocalcemia	Hypocalcemia	*p*
No of patients	87	71	16	
Female	63	48 (67%)	15 (94%)	<0.05 *
Age (average, range)	47.9 (18–79)	48.5 (21–77)	45.3 (18–79)	NS
PTH preop, pg/mL (range)	73.9 (26.0–218.0)	78.2 (29.5–218.0)	65.1 (26.0–127.0)	NS
pmol/L (range)	7.84 (2.76–23.12)	8.29 (3.13–23.21)	6.90 (2.76–13.47)	
Ca preop, mg/dL (range)	9.6 (8.6–10.7)	9.7 (8.6–10.7)	9.4 (8.6–10.0)	NS
mmol/L (range)	2.4 (2.15–2.67)	2.42 (2.15–2.67)	2.35 (2.15–2.5)	
Albumin preop, mg/dL (range)	4.4 (3.5–5.3)	4.4 (3.5–5.3)	4.3 (3.8–4.7)	NS
Vitamin D, preop (range)	19.5 (10.5–38.2)	21.4 (16.2–27.6)	19.0 (10.5–38.2)	NS
PTH 10 min, pg/mL (range)	50.7 (4.3–196.0)	56.8 (10.5–196.0)	23.9 (4.3–89.3)	<0.0001
pmol/L (range)	5.38 (0.46–20.79)	6.02 (1.11–20.79)	2.54 (0.46–9.47)	
Ca 10 min, mg/dL (range)	9.0 (7.9–10.2)	9.1 (8.2–10.2)	8.5 (7.9–9.1)	<0.0001
mmol/L (range)	2.25 (1.97–2.54)	2.27 (2.05–2.54)	2.12 (1.97–2.27)	
PTH 4 h, pg/mL (range)	50.6 (1.9–210.0)	57.8 (10.0–210.0)	19.5 (1.9–50.3)	<0.0001
pmol/L (range)	5.37 (0.20–22.27)	6.13 (1.06–22.27)	2.07 (0.20–5.33)	
Ca 4 h, mg/dL (range)	9.0 (8.1–10.4)	9.0 (8.1–10.4)	8.5 (8.2–9.2)	<0.0001
mmol/L (range)	2.25 (2.02–2.59)	2.25 (2.02–2.59)	2.12 (2.05–2.30)	
PTH 10 min/preop (average, range)	0.68 (0.127–1.905)	0.7 (0.185–1.905)	0.4 (0.127–0.911)	<0.0001
PTH 4 h/preop (average, range)	0.67 (0.043–1.336)	0.8 (0.200–1.336)	0.3 (0.043–0.729)	<0.0001
Ca 10 min/preop (average, range)	0.93 (0.81–1.03)	0.94 (0.85–1.03)	0.91 (0.81–0.99)	NS
Ca 4 h/preop (average, range)	0.93 (0.77–1.02)	0.93 (0.77–1.02)	0.91 (0.84–0.98)	NS

NS Non-significant, * Non-significant based on Bonferroni correction for multiple comparisons (*p* should be <0.001).

**Table 2:** Performance of the studied parameters for the identification of patients at risk for postoperative hypocalcemia.

	AUC	AUC SD	Cutoff	Accuracy	Sensitivity	Specificity	FNR	FPR	PPV	NPV	LR+	LR−	DOR	Youden’s J Statistic
PTH pre	0.755	0.073	51.650	0.828	0.313	**0.944**	0.688	**0.056**	0.556	0.859	5.547	0.729	7.614	0.256
Ca pre	0.742	0.077	9.350	0.816	0.625	0.859	0.375	0.141	0.500	0.911	4.438	0.436	10.167	0.484
PTH 10 min	0.821	0.069	23.550	0.885	0.688	0.930	0.313	0.070	0.688	0.930	**9.763**	0.336	29.041	0.617
Ca 10 min	0.851	0.047	8.850	0.713	0.875	0.676	**0.125**	0.070	0.378	0.960	2.701	0.185	14.610	0.551
PTH 4 h	0.893	0.045	30.500	0.851	0.750	0.873	0.250	0.127	0.571	0.939	3.701	0.286	12.927	0.623
Ca 4 h	0.834	0.050	8.850	0.724	**0.938**	0.676	0.063	0.324	0.395	**0.980**	4.701	**0.092**	**50.855**	0.614
Ca 10 min/Ca pre	0.647	0.084	0.905	0.793	0.563	0.845	0.438	0.155	0.450	0.896	5.701	0.518	11.013	0.408
Ca 4 h/Ca pre	0.649	0.066	0.936	0.621	0.813	0.578	0.188	0.423	0.302	0.932	6.701	0.325	20.639	0.390
PTH 10 min/PTH pre	0.838	0.060	0.406	0.839	0.688	0.873	0.313	0.127	0.550	0.925	7.701	0.358	21.519	0.561
PTH 4 h/PTH pre	0.892	0.045	0.385	**0.908**	0.750	**0.944**	0.250	**0.056**	**0.750**	0.944	8.701	0.265	32.845	**0.694**

AUC area under the curve, SD standard deviation, FNR false negative rate, FPR false positive rate, PPV positive predictive value, NPV negative predictive value, LR+ positive likelihood ratio, LR− negative likelihood ration, DOR diagnostic odds ratio. bold depicts the top value.

**Table 3:** Parameters retained in the model as significant in the binary logistic regression with the Forward Likelihood Ratio Method.

Variable	Regression Weights	Significance	Odds Ratio	95% CI
PTH preop	−0.030	0.021	0.971	0.942–1.000
Ca 4 h	−3.592	0.002	0.028	0.001–0.576
Ratio PTH 4 h/preop	−6.414	0.010	0.002	0.000–0.102
