# Correction: Daskalaki et al. Early Postoperative Parathormone and Calcium as Prognostic Factors for Postoperative Hypocalcemia. *J. Clin. Med.* 2022, *11*, 2389

**DOI:** 10.3390/jcm11247280

**Published:** 2022-12-08

**Authors:** Anna Daskalaki, Sofia Xenaki, Konstantinos Lasithiotakis, Alexandros Chrysos, Marilena Kampa, George Notas, Emmanuel Chrysos

**Affiliations:** 1 Department of General Surgery, University Hospital of Heraklion, 715 00 Heraklion, Greece; 2 Laboratory of Experimental Endocrinology, Department of Laboratory Medicine, University of Crete School of Medicine, 715 00 Giofirakia, Greece; 3 Department of General, Visceral and Transplantation Surgery, University Clinic RWTH Aachen, 52074 Aachen, Germany; 4 Department of Emergency Medicine, University Hospital of Heraklion, 715 00 Heraklion, Greece

## Additional Affiliations

In the published article [1], there was an error regarding the affiliation for Anna Daskalaki. In addition to affiliation 1, affiliation 2 should be added and the rest of the affiliations reordered. The correct format is as follows:

Anna Daskalaki ^1,2,^*, Sofia Xenaki ^1^, Konstantinos Lasithiotakis ^1^, Alexandros Chrysos ^1,3^, Marilena Kampa ^2^, George Notas ^2,4,^* and Emmanuel Chrysos ^1^

^1^ Department of General Surgery, University Hospital of Heraklion, 715 00 Heraklion, Greece

^2^ Laboratory of Experimental Endocrinology, Department of Laboratory Medicine, University of Crete School of Medicine, 715 00 Giofirakia, Greece

^3^ Department of General, Visceral and Transplantation Surgery, University Clinic RWTH Aachen, 52074 Aachen, Germany

^4^ Department of Emergency Medicine, University Hospital of Heraklion, 715 00 Heraklion, Greece

* Correspondence: daskalaki.a@hotmail.com (A.D.); gnotas@uoc.gr (G.N.); Tel.: +30-28-1039-2416 (A.D.); +30-28-1039-4556 (G.N.)

The authors apologize for any inconvenience caused and confirm that the scientific conclusions are unaffected by this change. This correction was approved by the Academic Editor. The original publication has also been updated.

## References

[1] Daskalaki A., Xenaki S., Lasithiotakis K., Chrysos A., Kampa M., Notas G., Chrysos E. (2022). Early Postoperative Parathormone and Calcium as Prognostic Factors for Postoperative Hypocalcemia. J. Clin. Med..

